# Generation of ‘designer erythroblasts’ lacking one or more blood group systems from CRISPR/Cas9 gene‐edited human‐induced pluripotent stem cells

**DOI:** 10.1111/jcmm.16872

**Published:** 2021-09-21

**Authors:** Priyanka Pandey, Nanyan Zhang, Brian R. Curtis, Peter J. Newman, Gregory A. Denomme

**Affiliations:** ^1^ Versiti Blood Research Institute Milwaukee WI USA; ^2^ Diagnostic Laboratories Versiti Blood Center of Wisconsin Milwaukee WI USA; ^3^ Departments of Pharmacology and Cellular Biology Medical College of Wisconsin Milwaukee WI USA

**Keywords:** alloimmunization, antibody identification, blood group systems, CRISPR, erythroblasts, hiPSC

## Abstract

Despite the recent advancements in transfusion medicine, red blood cell (RBC) alloimmunization remains a challenge for multiparous women and chronically transfused patients. At times, diagnostic laboratories depend on difficult‐to‐procure rare reagent RBCs for the identification of different alloantibodies in such subjects. We have addressed this issue by developing erythroblasts with custom phenotypes (Rh null, GPB null and Kx null/Kell low) using CRISPR/Cas9 gene‐editing of a human induced pluripotent stem cell (hiPSC) parent line (OT1‐1) for the blood group system genes: *RHAG*, *GYPB* and *XK*. Guide RNAs were cloned into Cas9‐puromycin expression vector and transfected into OT1‐1. Genotyping was performed to select puromycin‐resistant hiPSC KOs. CRISPR/Cas9 gene‐editing resulted in the successful generation of three KO lines, *RHAG* KO, *GYPB* KO and *XK* KO. The OT1‐1 cell line, as well as the three KO hiPSC lines, were differentiated into CD34^+^CD41^+^CD235ab^+^ hematopoietic progenitor cells (HPCs) and subsequently to erythroblasts. Native OT1‐1 erythroblasts were positive for the expression of Rh, MNS, Kell and H blood group systems. Differentiation of *RHAG* KO, *GYPB* KO and *XK* KO resulted in the formation of Rh null, GPB null and Kx null/Kell low erythroblasts, respectively. OT1‐1 as well as the three KO erythroblasts remained positive for RBC markers—CD71 and BAND3. Erythroblasts were mostly at the polychromatic/ orthochromatic stage of differentiation. Up to ~400‐fold increase in erythroblasts derived from HPCs was observed. The availability of custom erythroblasts generated from CRISPR/Cas9 gene‐edited hiPSC should be a useful addition to the tools currently used for the detection of clinically important red cell alloantibodies.

## 1 INTRODUCTION

Erythrocyte alloimmunization is a problem commonly seen in multiparous women and recipients of repeated transfusions, including patients with sickle cell anaemia, thalassemia, myelodysplastic syndrome and certain cancers.^1^, ^2^, ^3^ In diagnostic laboratories, red blood cell (RBC) alloantibody identification panels, consisting of blood group O RBCs phenotyped for common antigens from clinically relevant blood group systems, are routinely employed to evaluate the presence of clinically relevant alloantibodies in plasma or serum.

At times, alloantibodies are directed against a high‐prevalence antigen (present on RBCs of >99% population) for instance, anti‐Rh17: directed against the Rh17 antigen of the Rh blood group system,^4^ anti‐U: directed against the U antigen residing on Glycophorin B (GPB) of the MNS blood group system,^5^, ^6^ or anti‐Ku directed against Kell glycoprotein of the Kell blood group system.^7^ Serologic testing of alloantibodies against high‐prevalence antigens is difficult because they give a positive reaction with all cells in the panel.^8^ To confirm the specificity of such alloantibodies, diagnostic laboratories require antigen‐negative reagent RBCs like Rh null, GPB null or Kx null/Kell low. Though they would be the useful diagnostic reagents, RBCs negative for high‐prevalence antigens are not normally represented in antibody identification panels.

To address challenging alloantibody identifications, diagnostic laboratories currently use difficult‐to‐procure RBCs donated by blood donors with the desired RBC phenotype. It is not easy; however, to find and recruit such donors on a consistent basis. Diagnostic laboratories commonly freeze and store rare RBCs for future use, since they have a limited life span (~35 days). It is also resource‐intensive to find a consented donor with a required red cell antigen phenotype. Given the current constraints, blood donors are a finite resource for such cells.^9^, ^10^

As a means to address these problems, we have created ‘designer erythroblasts’ with custom phenotypes from a human induced pluripotent stem cell (hiPSC) line.^11^ The hiPSC were chosen as source material because they provide an infinite source of hematopoietic progenitor cells (HPCs), and upon further differentiation, RBCs.^12^ The Rh, MNS and Kx blood group systems were first targeted for the gene knockout studies, with a focus on the ability to generate erythroblasts that could be used to identify alloantibodies to high‐prevalence alloantigens in these systems.^13^

The following genes were chosen to knockout the selected blood group systems: (1) Rh blood group system Rh‐associated glycoprotein (*RHAG*, chromosome 6) gene is responsible for the red cell surface expression of the RhD and RhCE proteins.^14^, ^15^ (2) MNS blood group system Glycophorin B (*GYPB*, chromosome 4) gene is responsible for the red cell surface expression of S, s and U antigens (located on GPB protein).^16^, ^17^, ^18^, ^19^ (3) Kx blood group system *XK* gene (X chromosome) is responsible for the red cell surface expression of Kx protein. Loss of Kx leads to the McLeod neuroacanthocytosis syndrome, characterized by RBCs with abnormal morphology.^20^ Kx is attached to Kell glycoprotein by a single disulphide linkage; its absence leads to diminished expression of Kell antigen. Absence of RhAG, GPB and Kx proteins from the red cell surface results in Rh null, GPB null and Kx null/Kell low phenotypes, respectively.

The above‐mentioned RBC null phenotypes are observed infrequently amongst blood donors. Lab‐generated erythroblasts with the rare phenotypes Rh null, GPB null and Kx null/Kell low have the potential to provide difficult‐to‐procure reagent red cell controls in clinical diagnostic laboratories.

## 2 MATERIALS AND METHODS

### 2.1 Guide RNA design

Guide RNAs (gRNA) were designed using online portals (crispr.mit.edu and IDT’s custom design tool). Guides were designed to produce a large deletion so as to facilitate genotyping. Exons were selected that encode key regions of the protein in question. To ensure the loss of function for a gene, at least one gRNA was targeted to an exonic region. Guides were annealed per conditions detailed in Table S1. The strategy to clone gRNAs with BbsI overhangs into Cas9 expression vectors was adapted from Ran et al.^21^

### 2.2 Antibodies

Rat monoclonal anti‐SSEA3 (Clone MC‐631) along with the following mouse monoclonal antibodies were used: anti‐SSEA3, anti‐SSEA4 (Clone MC‐813–70), anti‐CD34 (Clone V MA27), anti‐CD41 (Integrin GPIIb, Clone IV P38), anti‐CD235ab (Glycophorin A/B, Clone VII 70299), anti‐CD235a (Glycophorin A, Clone VII 70312), anti‐CD71 (transferrin receptor, Clone A015) (Biolegend, San Diego, CA); anti‐CD238 (Kell, Clone BRIC 203), anti‐CD234 (Duffy, Clone 2C3) (BD Biosciences, San Jose, CA); anti‐RhAG (Clone LA1818) (kindly provided by Prof Ellen van der Schoot, Sanquin, Amsterdam, Netherlands); anti‐RhD/CE (Clone BRIC69, Fisher Scientific); anti‐U (Glycophorin B, Human plasma, Versiti‐Wisconsin); anti‐CD233 (BAND3, Clone BRIC6, American Research Products, Palos Verdes Estates, CA); anti‐CD173 (H antigen, Clone BRIC 231, MyBioSource, San Diego, CA). Secondary antibodies used were as follows: monoclonal APC‐conjugated Rat anti‐mouse IgG_1_ (Clone RMG1‐1, Biolegend) and polyclonal APC‐conjugated goat anti‐human IgG (Fab) (RRID: AB_2337691, Jackson Laboratories, Bar Harbor, ME).

### 2.3 Cytokines

Recombinant human proteins were used as follows: bFGF, BMP‐4, VEGF, SCF (R&D Systems, Minneapolis, MN), FLT3 Ligand (Fisher Scientific, Waltham, MA), Erythropoietin human (Sigma Aldrich, St. Louis, MO) and IL‐3 (STEMCELL Technologies, Vancouver, Canada). Small molecules CHIR 99021 (R&D Systems) and XAV939 (Sigma Aldrich) were used.

### 2.4 K562 cells

Optimization experiments to identify successful gRNA combinations to knockout the selected genes were performed using K562 cells. Briefly, cloning was performed by ligating annealed‐guides into BbsI digested GFP‐Cas9 expression vector pX458 (one guide/vector) using a quick ligation kit (New England Biolabs, Ipswich, MA, USA) and transformed into competent *E. coli* cells (Thermo Fisher Scientific, Waltham, MA, USA). Sequencing was performed to confirm the clones. Various gRNA combinations were electroporated into K562 cells using Cell Line Nucleofector Kit V (Lonza, Basel, Switzerland). The cells were grown overnight at 37°C/5%CO_2_. GFP‐positive K562 cells were sorted (BD FACSAria III Cell Sorter), collected, and cultured for 5–7 days. A total of nine (*RHAG*), three (*GYPB*) and four (*XK*) gRNA combinations were tested. Genotyping and flow cytometry were performed to confirm the gene deletion and subsequent loss of function (data are not shown).

### 2.5 hiPSC knockouts

Combinatorial gRNAs identified from the K562 experiments were cloned into the Cas9/puromycin expression vector, pX459V2. Pre‐screening in K562 cells was performed because hiPSC are adherent cells that grow in large aggregate colonies, making hiPSCs less compatible with cell sorter‐based GFP‐selection, a process that is easily performed using suspension K562 cells. For transfection, the parent hiPSC cell line (OT1‐1^11^) was plated at high density such that the cells became 50%–70% confluent within two days. On the day of transfection, OT1‐1 was incubated in Stemflex medium (Gibco, Fisher Scientific) containing the ROCK inhibitor (Y27632, STEMCELL technologies) for 2 h. Selected gRNA combinations (Table S1) were transfected into OT1‐1 using a P3 Primary Cell 4D‐Nucleofector X Kit S (Lonza, Basel, Switzerland), per manufacturer's protocol. Three separate transfections were performed using OT1‐1 to create knockout (KO) hiPSC lines, *RHAG* KO, *GYPB* KO and *XK* KO. Non‐transfected OT1‐1 served as a negative control. Transfected OT1‐1 was selected for puromycin‐resistant hiPSC clones by incubating them in media containing puromycin for a period of 24–36 h at 37°C/5%CO_2_/4%O_2_. Cells were replenished with media without puromycin and allowed to grow for a period of 5–10 days, with media changes every day until the colonies were large enough to be picked. Single‐cell hiPSC clones were processed into small clumps using a 26‐gauge needle and cultured into 24‐well plates. At least 24 colonies were picked per gene knockout. Genotyping was performed to confirm the knockouts. Genotyping conditions have been detailed in Table S2.

### 2.6 Differentiation of hiPSC to HPC to erythroblasts

**FIGURE 1:**
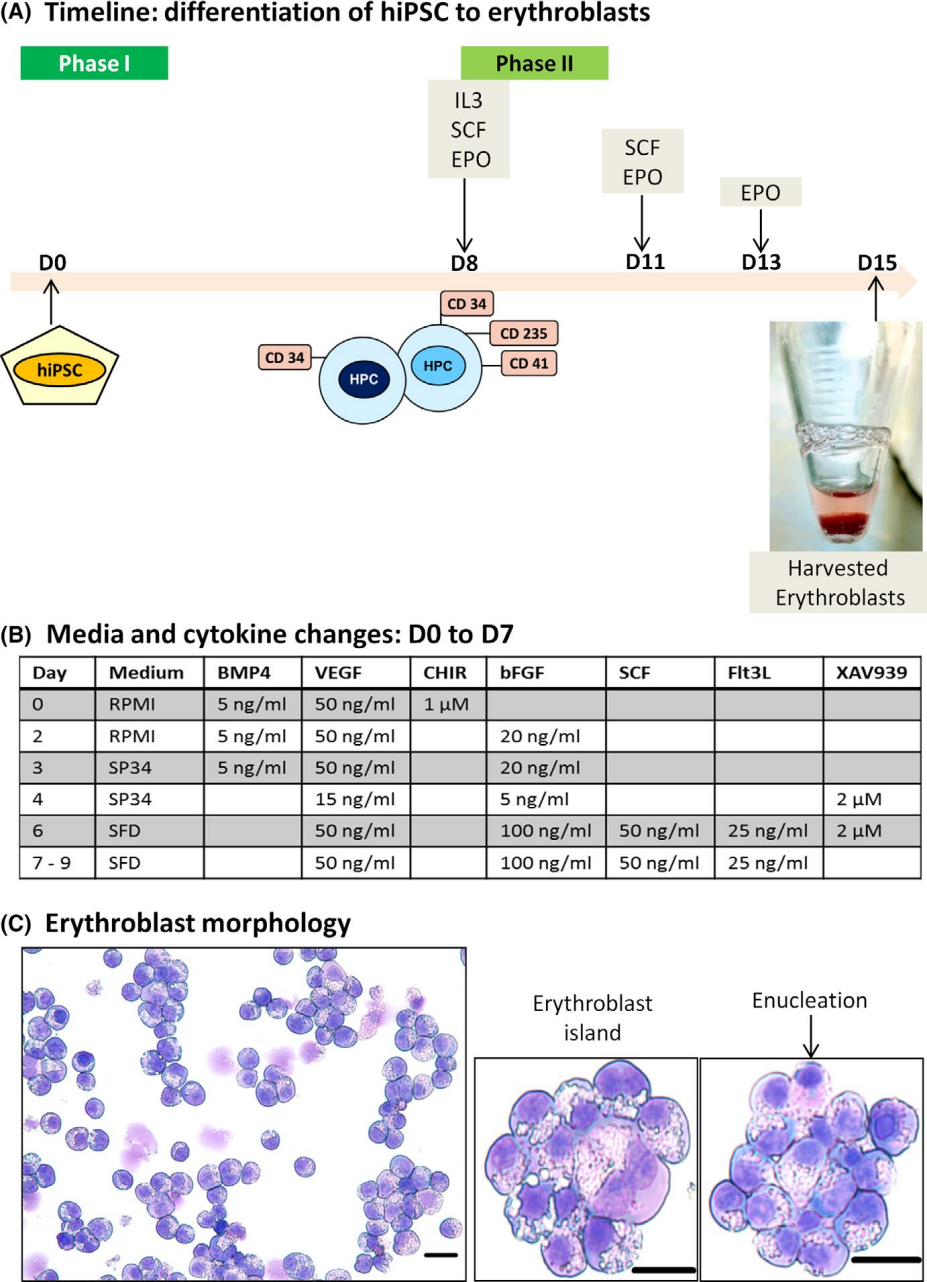
(A) Differentiation of hiPSC to HPC (Phase I) and erythroblasts (Phase II). At the end of phase I, hematopoietic progenitors were observed displaying CD34 (stem‐cell marker), erythro‐megakaryocytic markers, CD235ab and CD41. These progenitor cells were further seeded and induced to generate erythroblasts in phase II. (B) Stepwise (by day) media and cytokine changes. Differentiation of hiPSC to HPC from D0 to D7 required regular media changes and different cytokine stimulations. The HPCs can be expanded up to 9 days (C) Erythroblast morphology. Most of the erythroblasts were either polychromatic or orthochromatic. Few erythroblast islands were observed consisting of macrophage‐like centre and erythroblast rosettes. A minor population of enucleated cells or cells about to enucleate was also observed. Magnification: 400×. Scale bar: 20 µm. bFGF, basic fibroblast growth factor; BMP‐4, bone morphogenetic protein 4; CHIR99021, GSK3β inhibitor; D, day; EPO, erythropoietin (5 U/ml); Flt3L, FMS‐like tyrosine kinase 3 ligand; IL3, interleukin 3 (5 ng/ml); SCF, stem cell factor (50 ng/ml); SCF, stem cell factor; VEGF, vascular endothelial growth factor; XAV939, Wnt‐signalling inhibitor

OT1‐1 as well *RHAG* KO, *GYPB* KO and *XK* KO hiPSC lines were differentiated to HPCs using a protocol from Mills et al.^22^ and Zhang et al.^11^ Differentiation of hiPSCs to HPCs (Phase I) and subsequently to erythroblasts (Phase II) is divided into two phases (Figure 1A). Briefly, in Phase I hiPSCs were seeded at a high density on a Matrigel‐coated 6‐well plate, so that the cells achieve ~70% confluence within 2 days. On day 0, before starting the process of differentiation, one well of the 6‐well plate containing hiPSCs was sacrificed to assess the pluripotency status of hiPSCs using SSEA3 and SSEA4. Media and cytokine changes were made per Figure 1B. On day 8, loosely bound HPCs were collected by vigorous pipetting and evaluated for hematopoietic and stem cell markers including CD41, CD235ab and CD34. In Phase II, harvested HPCs were seeded in ultra‐low attachment 6‐well culture plates (Corning, Corning, NY) at a density of 10^5^ cells/well containing SFEM‐II media (STEMCELL Technologies, Vancouver, Canada) supplemented with IL‐3 (5 ng/ml), SCF (50 ng/ml) and EPO (5 U/ml) and incubated for 3 days. Later, fresh SFEM‐II media supplemented with SCF (50 ng/ml) and EPO (5 U/ml) was added, and the cells were incubated for 2 days. On day 13, fresh SFEM‐II media supplemented with EPO (5 U/ml) was added to the wells. During phase II, media changes were made in a way so as not to disturb the seeded cells with almost ninety per cent old media being removed each time. On day 15, erythroblasts were harvested for further analysis.

### 2.7 Flow cytometric evaluation of red cells antigens

Erythroblasts were washed once and resuspended in phosphate buffer saline (PBS) containing 1% bovine serum albumin (BSA). Commercial antibodies were used per manufacturer's instructions. Anti‐RhAG (1:20 dilution), and anti‐U (Titre = 1:80, 5 µl undiluted plasma) per 10^6^ cells in a 100 µl reaction were used. Appropriate isotype and secondary antibody controls were employed. All primary and secondary antibody incubations were performed at 4°C for 30 min. Flow cytometry was performed on BD LSR II Flow Cytometer. Erythroblasts were evaluated for antigens from blood group systems including Rh, MNS, Kell, Duffy and H. The cells were additionally evaluated for other markers including RhAG, CD71 (transferrin receptor) and BAND3 (integral red cell membrane protein).

### 2.8 Cytospin preparation

Erythroblasts were washed once and resuspended in PBS containing 30% heat‐inactivated foetal bovine serum. Cytospin preparations were made with 10^6^ erythroblasts per glass slide (1000 rpm/5 min). The slides were dried for 5 min in a laminar flow hood and fixed in cold methanol for 10 min at −20°C. The slides were dried and stained with Wright‐Giemsa stain (Sigma Aldrich, St. Louis, MO, USA) according to the manufacturer's protocol. Cytospins were imaged at 400X using a Nikon Eclipse E600 microscope.

## 3 RESULTS

### 3.1 Generation of erythroblasts from hiPSC

**FIGURE 2:**
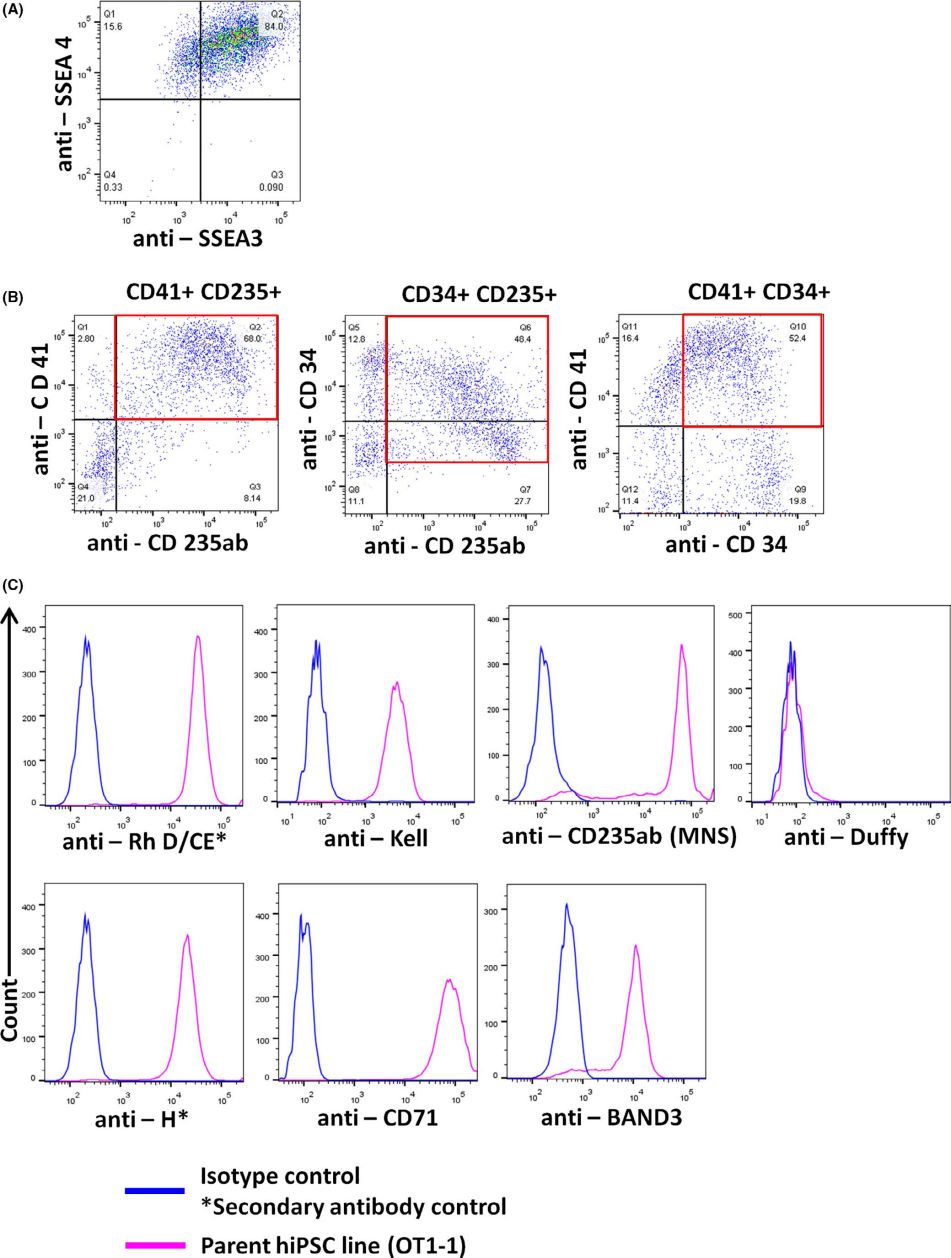
Cell surface antigen presentation. (A) On day 0, hiPSC stained positive for pluripotency markers SSEA3/4. (B) On day 8, hematopoietic progenitors were positive for CD34, CD41 and CD235ab markers. (C) On day 15, erythroblasts were harvested and observed to be positive for Rh, MNS, Kell, H blood group system antigens and the red cell surface markers CD71 (transferrin receptor) and BAND3 (integral red cell membrane protein). Erythroblasts were negative for Duffy blood group system concordant with the hiPSC donor genotype (*FYA*/*FYB* negative)

The hiPSCs were positive for SSEA3/4, confirming normal pluripotency status (Figure 2A). On Day 8 of culture, a heterogeneous population of HPCs was observed; all the cells were CD34^+^ and a 50%–70% population was CD34^+^ CD235ab^+^ CD41^+^ (Figure 2B). Up to ~400‐fold expansion was observed from the HPC to erythroblast stage (data are not shown). Erythroblasts from the parent OT1‐1 line expressed RhD/CE, MNS (GYPA/B, CD235ab), Kell, H (precursor antigen to ABO antigens), BAND3 (integral red cell membrane protein) and CD71 (transferrin receptor). The cells were negative for the Duffy antigen, consistent with the *FYA*/*FYB* negative genotype of the donor (Figure 2C).

### 3.2 Erythroblast morphology

Most of the erythroblasts were either at the polychromatic or orthochromatic stage of differentiation, with vacuoles present in the cytoplasm. A few cells in the process of enucleation were observed, amongst mostly nucleated erythroid progenitor cells. Microscopic images revealed the formation of erythroblast islands consisting of a macrophage‐like centre and erythroblasts in rosettes (Figure 1C).

### 3.3 Confirmation of hiPSC KO lines

**FIGURE 3:**
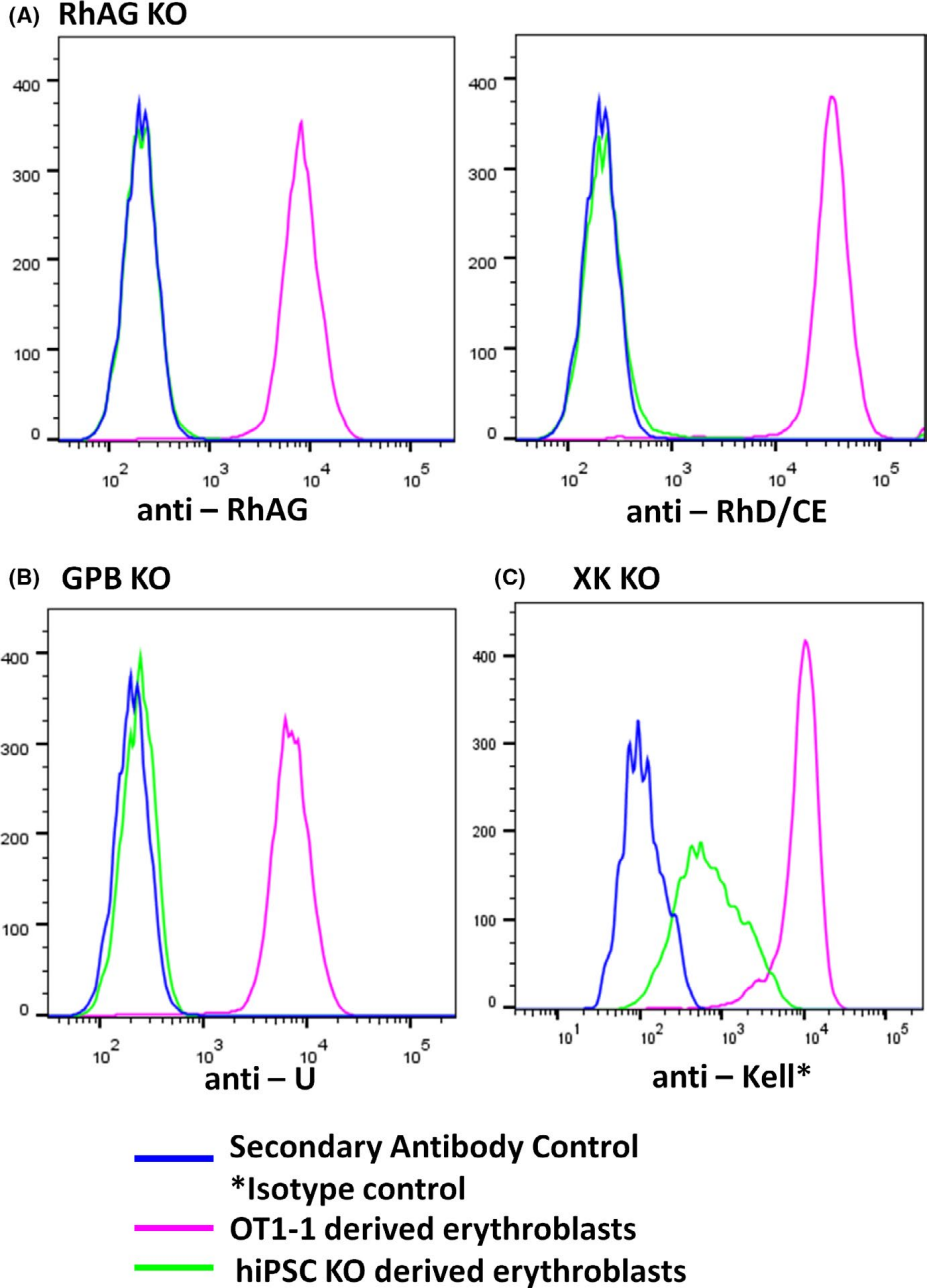
CRISPR/Cas9 gene‐edited erythroblasts surface antigen presentation. Complete loss of RhAG, RhD/CE, GPB protein expression was confirmed for RhAG, RhD/CE (A) and GPB (B) erythroblasts. Anti‐U was used to confirm GPB deletion. Kell glycoprotein expression was evaluated as a proxy for XK knockout erythroblasts. Kell expression (C) was considerably diminished consistently with the lack of Kx expression. KO, knockout

Genotyping confirmed deletion of targeted segments of genes, *RHAG*, *GYPB* and *XK* with the presence of the expected shorter band consistent with the CRISPR/Cas9 knockout strategy (Figure S1A–C). Expression of RhAG KO erythroblasts revealed successful generation of Rh null erythroblasts, that is, the complete absence of RhAG and RhD/CE proteins from the erythroblasts surface, evident by complete overlap of KO cell peak with the control (Figure 3A). Expression of GPB KO evaluated using anti‐U (from a donor plasma) revealed successful generation of GPB null erythroblasts with a complete overlap of KO cell peak with the control (Figure 3B). Due to the unavailability of anti‐Kx, expression of *XK* KO derived erythroblasts was evaluated using anti‐Kell (Clone: BRIC203). A reduced expression of Kell antigens was observed on XK KO erythroblasts, as evidenced by an expected left shift of KO peak relative to OT1‐1 derived erythroblasts expression (Figure 3C). Karyotyping (performed by Wisconsin Diagnostic Laboratories, Milwaukee, WI) of all hiPSC KO lines confirmed normal chromosome copy number and banding pattern (data are not shown). Erythroblasts from hiPSC KO lines also displayed normal expression of CD71 and BAND3 (data are not shown).

## 4 DISCUSSION

A desire to manipulate human RBCs was first attempted by Goldstein and colleagues who demonstrated the successful enzymatic conversion of Group B RBCs to Group O and subsequent transfusion to Group O/A individuals.^23^, ^24^, ^25^ Since then, other RBC moieties have been manipulated in the laboratory.

Red cell alloimmunization is a problem that quite often complicates the provision of compatible blood for chronic transfusion recipients and multiparous women.^26^, ^27^, ^28^ Diagnostic laboratories employ reagent RBCs with desired antigenic profiles (obtained from rare specialized donors) to ascertain the alloantibody specificities, so that compatible blood can be found. Advancements have been made towards employing reagent RBCs that are not only serologically typed but also genotyped to increase the scope of antibody identification panels.^29^, ^30^, ^31^, ^32^ Currently, reagent red cells are obtained from whole blood donations. Specific RBC antigen profiles are necessary to optimize the number of reagent red cells needed for antibody identification studies by clinical labs. Historically, antibody‐based phenotyping methods were used to identify suitable blood. By 2005, blood centres developed broad red cell genotyping methods using synthetic reagents to find suitable blood for manufacture into reagent red cells.^33^ Many more genotypes could be considerly compared to antibody‐based phenotyping methods, and with better accuracy.^34^ By 2015, the serologic approach to screen blood was abandoned with major efficiencies made in the screening process as mass‐scale approaches were refined.^32^, ^35^ Now, designer RBCs production opens up the opportunity to create novel reagent red cells that are extremely rare or possibly non‐existent amongst humans.

Still, at times reagent RBCs need to be treated with either proteolytic enzymes, chemicals or PEG^36^ in order to mask the effect of high‐prevalence antigens, to aid the process of antibody identifications.^37^ For instance, to avoid pan‐agglutination of antibody identification panel red cells, sera of DARA‐treated myeloma patients require reagent RBCs to be treated with DTT. Such harsh treatments leave RBCs suitable for only one‐time use and at times disrupts the surface expression of other blood group antigens. For instance, DTT treatment denatures Kell glycoprotein expression, rendering reagent RBCs unsuitable to rule out alloantibodies to Kell blood group system antigens.^38^ Also, such processes are time‐consuming and labour‐intensive.

Despite such a robust system, red cells with extremely rare phenotypes like Rh null, GPB null or Kx null can be required to identify such specificities using antibody identification panels. In our study, we have tried to address this issue by CRISPR‐Cas9 gene‐editing of a well‐characterized, HLA Class I‐negative immortalized hiPSC line in order to generate Designer Red cells having custom cell‐surface phenotypes. Alloantibodies against Rh, Kell and MNS blood group systems are commonly observed in patients.^39^ Hence, we decided to work on deleting blood group systems, (1) Rh/RhAG, (2) MNS (GPB) and (3) Kx/Kell, as the resulting erythroblasts would be valuable typing reagents for the detection of high‐prevalence alloantibodies.

CRISPR‐Cas9 gene‐editing can be performed by either targeting a gene with a single guide RNA or two guide RNAs. We employed a two guide—one gene strategy to knockout blood group system genes in hiPSC. This was done to ease the process of genotyping hiPSC clones as deletion of a large fragment due to the action of two guide RNAs resulted in a shorter fragment upon PCR of the knockout hiPSC cell line. The transfection of hiPSC with two‐guide RNA constructs increased the probability for the success of this gene‐editing approach. Also, because native hiPSC do not express red cell blood group antigens so they could not be directly screened using flow cytometry like K562 cells. Optimization experiments were performed in K562 cells to identify the gRNA combinations that resulted in deletion of the gene in question. The K562 human erythroleukaemia cell line was chosen as it has a proerythroblast phenotype and is amenable to flow cytometric detection of cell surface antigens. Once the working gRNA combinations were established, final gene‐editing was carried out in hiPSCs.

For *RHAG* and *XK* KO hiPSC, we were able to obtain a single short band demonstrating the desired gene‐editing effect. Flow cytometric evaluation of hiPSC gene knockout erythroblasts demonstrated complete loss of RhAG and RhD/CE proteins. Also, Kell antigen expression was remarkably reduced from the XK KO erythroblasts, consistent with previous findings in humans.^40^ For *GYPB* KO, initial evaluation was the presence of mixed clones with two band patterns; apparent wild type (long band) and confirmed KO (short band). A few *GYPB* KO hiPSC mixed clones were differentiated from erythroblasts. The absence of GPB expression in spite of the presence of a longer (apparent wild type) band in *GYPB* KO construct suggests that in some cells only a single gRNA might have worked.

The hiPSC were evaluated for the pluripotency markers SSEA3/4 before starting the process of differentiation. Differentiation of hiPSC with a collection of cytokines and media changes over 8–9 days resulted in the production of a heterogeneous population of hematopoietic progenitors with most of the progenitors positive for CD34, CD235ab and CD41. Whilst CD235 is an erythroid‐specific marker and CD41 is a megakaryocytic lineage‐specific marker, both CD235 and CD41 are the markers for primitive hematopoietic progenitors.^22^, ^41^ The progenitors were differentiated towards erythroid lineage using IL3, SCF and EPO. IL3 was removed after three days and SCF after five days as the cytokines are required only during the initial stages of erythropoiesis.^42^, ^43^, ^44^ Later, cells were incubated only with EPO in an attempt to induce terminal erythroid differentiation.^45^, ^46^ By day 14–15, substantial proliferation was observed, additionally, cells were haemoglobinized. The resulting erythroid cells were primarily polychromatic or orthochromatic erythroblasts varying in size from 6 µm‐8 µm. Vacuoles were observed to be present in the cytoplasm of the erythroblasts consistent with the findings of other studies on ex vivo generation of red cells.^47^, ^48^ Formation of vacuoles is a normal event in late erythropoiesis as erythroblasts prepare to enucleate. These vacuoles are lysosomal in nature and engulf cellular organelles including mitochondria that are later extruded at the reticulocyte stage.^49^ In vitro enucleation of erythroblasts is still a topic of active investigation. It is known that some enucleation occurs in cell culture^50^, ^51^; however, simulating the bone marrow environment ex vivo is still difficult to achieve. Efforts have been made by layering erythroblasts on feeder cells or macrophages to induce the process of enucleation but the results have not been very remarkable. Ongoing significant advancements in the field of stem cell biology as well as production of red cells should eventually pave the way for the generation of red cells in large numbers. Such advancements will also benefit the prospects of red cell therapeutics. Although successful production of mature red cells from hiPSC still remains a topic of active investigation; however, previous studies by Lapillone et al.,^12^ Kurita et al.,^52^ Dias et al.^53^ and Olivier et al.,^54^ have demonstrated successful production of erythroid cells from hiPSC. Lapillone et al., differentiated hiPSC to erythroid cells through embryoid body formation in a 26–27 day protocol.^12^ Kurita et al., have established an immortalized erythroid progenitor cell line from hiPSC co‐cultured with feeder cells to generate red cells,^52^ Dias et al., had co‐cultured hiPSC with feeder cells to generate hematopoietic progenitors with subsequent differentiation to erythroid cells.^53^ The method by Olivier et al., has utilized serum‐free and feeder‐free culture conditions to generate erythroid cells from hiPSC.^54^ The protocols established by the previous studies require numerous small molecules and cytokines in addition to EPO to generate erythroblasts.^55^, ^56^ In contrast, our protocol to generate flow cytometry‐ready erythroblasts from hiPSC derived hematopoietic progenitors is shorter (7–8 day protocol), utilizes only 3 cytokines (IL3, SCF, and EPO) producing high‐yield erythroblasts.

Past attempts at generating RBCs with rare phenotypes either utilized RhD negative donor hiPSCs^57^ or CRISPR/Cas9 editing of RhD negative donor hiPSC for RHCE,^58^ whilst our hiPSC was RhD/CE positive. By targeting *RHAG*, we successfully created an RBC with the rare Rh null phenotype resulting erythroblasts are devoid of all Rh antigens. We also successfully created GPB null and Kx null/Kell low erythroblasts. GPB null cells were evaluated using an anti‐U from human plasma, which were non‐reactive demonstrating that our lab‐generated erythroblasts could be used to detect other blood group alloantibodies in the presence of anti‐U. Also, the establishment of a hiPSC line capable of generating erythroblasts with the McLeod phenotype alleviates the ethical responsibility of identifying male blood donors with the potential for a disease (neuroacanthocytosis^59^) and of female carriers for this genetic disease.

Primary limitations of our study were low yield and poor enucleation rates of erythroblasts. Although we were able to observe a good expansion (~400 fold) of erythroblasts from the hematopoietic progenitor stage, the yield was not sufficient to run multiple tests using clinical lab methods. We anticipate individual nucleated erythroblasts (reagent erythroblasts) would behave similarly to the mature enucleated red cells (reagent red cells) in column agglutination assays. Immortalized hiPSC have a distinct advantage as a starting material for an unlimited source of progenitor cells. However, as observed by other investigators, enucleation amongst erythroblasts generated from hiPSC remains low.^54^, ^60^ As elaborated by Stella Chou,^61^ future ongoing significant advancements in the understanding of culture requirements could support the generation of mature red cells.^62^, ^63^ On a separate note, use of CD34^+^ cells^64^ or immortalization of CD34^+^ cells to generate erythroblasts/reticulocytes as shown by Trakarnsanga et al.^65^ could serve as an alternative to this approach.

In conclusion, we have successfully generated ‘designer erythroblasts’ with custom phenotypes (Rh null, GPB null and XK null/KEL low). The strategy could be applied to other blood group systems. Erythroid cells produced by CRISPR/Cas9 gene‐editing will serve to identify alloantibodies that otherwise cannot be confirmed using the natural polymorphisms of RBC antigens normally found in humans.

## CONFLICT OF INTEREST

The authors declare that there is no financial conflict of interest.

## AUTHOR CONTRIBUTIONS

**Priyanka Pandey:** Conceptualization (lead); Data curation (lead); Formal analysis (lead); Investigation (lead); Methodology (lead); Software (lead); Writing‐original draft (lead); Writing‐review & editing (lead). **Nanyan Zhang:** Methodology (supporting). **Brian R. Curtis:** Funding acquisition (equal); Resources (equal); Writing‐review & editing (equal). **Peter J. Newman:** Funding acquisition (equal); Methodology (equal); Project administration (equal); Resources (equal); Supervision (equal); Writing‐review & editing (equal). **Gregory A. Denomme:** Conceptualization (lead); Funding acquisition (lead); Investigation (lead); Methodology (lead); Project administration (lead); Resources (lead); Supervision (lead); Writing‐review & editing (lead).

## Supporting information

Fig S1

Table S1‐S2

## References

[1] Hendrickson JE , Tormey CA . Understanding red blood cell alloimmunization triggers. Hematology. 2016;2016(1):446‐451. 27913514 10.1182/asheducation-2016.1.446 PMC6142457

[2] Webb J , Delaney M . Red blood cell alloimmunization in the pregnant patient. Transfus Med Rev. 2018;32(4):213‐219. 30097223 10.1016/j.tmrv.2018.07.002

[3] Singhal D , Kutyna MM , Chhetri R , et al. Red cell alloimmunization is associated with development of autoantibodies and increased red cell transfusion requirements in myelodysplastic syndrome. Haematologica. 2017;102(12):2021‐2029. 28983058 10.3324/haematol.2017.175752 PMC5709101

[4] Dajak S , Ipavec N , Cuk M , et al. The outcome of hemolytic disease of the fetus and newborn caused by anti‐Rh17 antibody: analysis of three cases and review of the literature. Transfus Med Hemother. 2020;47(3):264‐271. 32595431 10.1159/000503012 PMC7315214

[5] Novaretti MCZ , Jens E , Pagliarini T , Bonifácio SL , Dorlhiac‐Llacer PE , Chamone DAF . Hemolytic disease of the newborn due to anti‐U. Rev Hosp Clin. 2003;58(6):320‐323. 10.1590/s0041-87812003000600006 14762491

[6] Rothman IK , Alter HJ , Strewler GJ . Delayed overt hemolytic transfusion reaction due to anti‐U antibody. Transfusion. 1976;16(4):357‐360. 951732 10.1046/j.1537-2995.1976.16476247058.x

[7] Mattaloni SM , Arnoni C , Céspedes R , et al. Clinical significance of an alloantibody against the kell blood group glycoprotein. Transfus Med Hemother. 2017;44(1):53‐57. 28275334 10.1159/000448381 PMC5318919

[8] Khan J , Delaney M . Transfusion support of minority patients: extended antigen donor typing and recruitment of minority blood donors. Transfus Med Hemother. 2018;45(4):271‐276. 30283277 10.1159/000491883 PMC6158592

[9] Hawksworth J , Satchwell TJ , Meinders M , et al. Enhancement of red blood cell transfusion compatibility using CRISPR‐mediated erythroblast gene editing. EMBO Mol Med. 2018;10(6):e8454. 29700043 10.15252/emmm.201708454 PMC5991592

[10] Kikuchi GO , Kurita R , Ogasawara K , et al. Application of immortalized human erythroid progenitor cell line in serologic tests to detect red blood cell alloantibodies. Transfusion. 2018;58(11):2675‐2682. 30180269 10.1111/trf.14840

[11] Zhang N , Newman PJ . Packaging functionally important plasma proteins into the alpha‐granules of human‐induced pluripotent stem cell‐derived megakaryocytes. J Tissue Eng Regen Med. 2019;13(2):244‐252. 30556311 10.1002/term.2785 PMC6742440

[12] Lapillonne H , Kobari L , Mazurier C , et al. Red blood cell generation from human induced pluripotent stem cells: perspectives for transfusion medicine. Haematologica. 2010;95(10):1651‐1659. 20494935 10.3324/haematol.2010.023556 PMC2948089

[13] Reid ME , Lomas‐Francis C , Olsson ML . The blood group antigen factsbook, 3rd ed. Elsevier/AP; 2012.

[14] Flegel WA . Molecular genetics and clinical applications for RH. Transfus Apher Sci. 2011;44(1):81‐91. 21277262 10.1016/j.transci.2010.12.013 PMC3042511

[15] Westhoff CM . The structure and function of the Rh antigen complex. Semin Hematol. 2007;44(1):42‐50. 17198846 10.1053/j.seminhematol.2006.09.010 PMC1831834

[16] Parsh BS . Hemolytic disease of the newborn due to anti S antibodies. J Natl Med Assoc. 2000;92(2):91‐93. 10800298 PMC2640537

[17] Pitan C , Syed A , Murphy W , Akinlabi O , Finan A . Anti‐S antibodies: an unusual cause of haemolytic disease of the fetus and newborn (HDFN). BMJ Case Rep. 2013;2013:bcr2012006547. 10.1136/bcr-2012-006547 PMC3604323 23291808

[18] Strindberg J , Lundahl J , Ajne G . Hemolytic disease of the fetus and newborn owing to anti‐U, successfully treated with repeated intrauterine transfusions. Immunohematology. 2013;29(2):51‐54. 24094236

[19] Ringressi A , Biagioni S , Mello G , Graziani G , Mecacci F . Anti‐U alloimmunisation in a pregnant woman from Niger. Blood Transfus. 2012;10(2):221‐224. 22337275 10.2450/2012.0049-11 PMC3320784

[20] Jung HH , Danek A , Walker RH , Frey BM , Gassner C . McLeod Neuroacanthocytosis Syndrome. In: Adam MP , Ardinger HH , Pagon RA , editors. GeneReviews((R)). University of Washington; 1993. 20301528

[21] Ran FA , Hsu PD , Wright J , Agarwala V , Scott DA , Zhang F . Genome engineering using the CRISPR‐Cas9 system. Nat Protoc. 2013;8(11):2281‐2308. 24157548 10.1038/nprot.2013.143 PMC3969860

[22] Mills JA , Paluru P , Weiss MJ , Gadue P , French DL . Hematopoietic differentiation of pluripotent stem cells in culture. Methods Mol Biol. 2014;1185:181‐194. 25062629 10.1007/978-1-4939-1133-2_12

[23] Goldstein J , Siviglia G , Hurst R , Lenny L , Reich L . Group B erythrocytes enzymatically converted to group O survive normally in A, B, and O individuals. Science. 1982;215(4529):168‐170. 6274021 10.1126/science.6274021

[24] Lenny LL , Hurst R , Goldstein J , Benjamin LJ , Jones RL . Single‐unit transfusions of RBC enzymatically converted from group B to group O to A and O normal volunteers. Blood. 1991;77(6):1383‐1388. 1848117

[25] Rahfeld P , Withers SG . Toward universal donor blood: enzymatic conversion of A and B to O type. J Biol Chem. 2020;295(2):325‐334. 31792054 10.1074/jbc.REV119.008164 PMC6956546

[26] Chou ST , Jackson T , Vege S , Smith‐Whitley K , Friedman DF , Westhoff CM . High prevalence of red blood cell alloimmunization in sickle cell disease despite transfusion from Rh‐matched minority donors. Blood. 2013;122(6):1062‐1071. 23723452 10.1182/blood-2013-03-490623

[27] Moise KJ . Red blood cell alloimmunization in pregnancy. Semin Hematol. 2005;42(3):169‐178. 16041667 10.1053/j.seminhematol.2005.04.007

[28] Sanz C , Nomdedeu M , Belkaid M , Martinez I , Nomdedeu B , Pereira A . Red blood cell alloimmunization in transfused patients with myelodysplastic syndrome or chronic myelomonocytic leukemia. Transfusion. 2013;53(4):710‐715. 22845746 10.1111/j.1537-2995.2012.03819.x

[29] Scharberg E , Rink G , Portegys J , et al. The impact of using genotyped reagent red blood cells in antibody identification. Transfus Med Hemother. 2018;45(4):218‐224. 30283270 10.1159/000491884 PMC6158584

[30] Casas J , Friedman DF , Jackson T , Vege S , Westhoff CM , Chou ST . Changing practice: red blood cell typing by molecular methods for patients with sickle cell disease. Transfusion. 2015;55(6 Pt 2):1388‐1393. 25573464 10.1111/trf.12987 PMC9003876

[31] Kappler‐Gratias S , Peyrard T , Rouger P , Le Pennec PY , Pham BN . Blood group genotyping by high‐throughput DNA analysis: application to the French panel of RBC reagents. Transfus Clin Biol. 2010;17(3):165‐167. 20655269 10.1016/j.tracli.2010.05.007

[32] Flegel WA , Gottschall JL , Denomme GA . Implementing mass‐scale red cell genotyping at a blood center. Transfusion. 2015;55(11):2610‐2615. quiz 2609. 26094790 10.1111/trf.13168 PMC4644091

[33] Denomme GA , Van Oene M . High‐throughput multiplex single‐nucleotide polymorphism analysis for red cell and platelet antigen genotypes. Transfusion. 2005;45(5):660‐666. 15847652 10.1111/j.1537-2995.2005.04365.x

[34] Krog GR , Rieneck K , Clausen FB , Steffensen R , Dziegiel MH . Blood group genotyping of blood donors: validation of a highly accurate routine method. Transfusion. 2019;59(10):3264‐3274. 31415105 10.1111/trf.15474

[35] Flegel WA , Gottschall JL , Denomme GA . Integration of red cell genotyping into the blood supply chain: a population‐based study. Lancet Haematol. 2015;2(7):e282‐e289. 26207259 10.1016/S2352-3026(15)00090-3 PMC4508019

[36] Bagnis C , Chiaroni J , Bailly P . Elimination of blood group antigens: hope and reality. Br J Haematol. 2011;152(4):392‐400. 21210778 10.1111/j.1365-2141.2010.08561.x

[37] Daniels G . Effect of enzymes on and chemical modifications of high‐frequency red cell antigens. Immunohematology. 1992;8(3):53‐57. 15946059

[38] Lancman G , Arinsburg S , Jhang J , et al. Blood transfusion management for patients treated with anti‐CD38 monoclonal antibodies. Front Immunol. 2018;9:2616. 30498492 10.3389/fimmu.2018.02616 PMC6249335

[39] Tormey CA , Hendrickson JE . Transfusion‐related red blood cell alloantibodies: induction and consequences. Blood. 2019;133(17):1821‐1830. 30808636 10.1182/blood-2018-08-833962 PMC6484385

[40] Murakami T , Abe D , Matsumoto H , et al. A patient with McLeod syndrome showing involvement of the central sensorimotor tracts for the legs. BMC Neurol. 2019;19(1):301. 31775676 10.1186/s12883-019-1526-9 PMC6882147

[41] Paluru P , Hudock KM , Cheng X , et al. The negative impact of Wnt signaling on megakaryocyte and primitive erythroid progenitors derived from human embryonic stem cells. Stem Cell Res. 2014;12(2):441‐451. 24412757 10.1016/j.scr.2013.12.003 PMC4048963

[42] Valent P , Büsche G , Theurl I , et al. Normal and pathological erythropoiesis in adults: from gene regulation to targeted treatment concepts. Haematologica. 2018;103(10):1593‐1603. 30076180 10.3324/haematol.2018.192518 PMC6165792

[43] Hattangadi SM , Wong P , Zhang L , Flygare J , Lodish HF . From stem cell to red cell: regulation of erythropoiesis at multiple levels by multiple proteins, RNAs, and chromatin modifications. Blood. 2011;118(24):6258‐6268. 21998215 10.1182/blood-2011-07-356006 PMC3236116

[44] Zeuner A , Francescangeli F , Signore M , et al. The Notch2‐Jagged1 interaction mediates stem cell factor signaling in erythropoiesis. Cell Death Differ. 2011;18(2):371‐380. 20829885 10.1038/cdd.2010.110 PMC3131890

[45] Malik J , Kim AR , Tyre KA , Cherukuri AR , Palis J . Erythropoietin critically regulates the terminal maturation of murine and human primitive erythroblasts. Haematologica. 2013;98(11):1778‐1787. 23894012 10.3324/haematol.2013.087361 PMC3815180

[46] Erickson N , Quesenberry PJ . Regulation of erythropoiesis. The role of growth factors. Med Clin North Am. 1992;76(3):745‐755. 1578968 10.1016/s0025-7125(16)30351-0

[47] Giarratana M‐C , Kobari L , Lapillonne H , et al. Ex vivo generation of fully mature human red blood cells from hematopoietic stem cells. Nat Biotechnol. 2005;23(1):69‐74. 15619619 10.1038/nbt1047

[48] Kobayashi I , Ubukawa K , Sugawara K , et al. Erythroblast enucleation is a dynein‐dependent process. Exp Hematol. 2016;44(4):247‐256.e212. 26724640 10.1016/j.exphem.2015.12.003

[49] Moras M , Lefevre SD , Ostuni MA . From erythroblasts to mature red blood cells: organelle clearance in mammals. Front Physiol. 2017;8:1076. 29311991 10.3389/fphys.2017.01076 PMC5742207

[50] McGrath KE . Red cell island dances: switching hands. Blood. 2014;123(25):3847‐3848. 24948619 10.1182/blood-2014-04-565531

[51] Toda S , Segawa K , Nagata S . MerTK‐mediated engulfment of pyrenocytes by central macrophages in erythroblastic islands. Blood. 2014;123(25):3963‐3971. 24659633 10.1182/blood-2014-01-547976

[52] Kurita R , Suda N , Sudo K , et al. Establishment of immortalized human erythroid progenitor cell lines able to produce enucleated red blood cells. PLoS One. 2013;8(3):e59890. 23533656 10.1371/journal.pone.0059890 PMC3606290

[53] Dias J , Gumenyuk M , Kang HyunJun , et al. Generation of red blood cells from human induced pluripotent stem cells. Stem Cells Dev. 2011;20(9):1639‐1647. 21434814 10.1089/scd.2011.0078 PMC3161101

[54] Olivier EN , Marenah L , McCahill A , Condie A , Cowan S , Mountford JC . High‐efficiency serum‐free feeder‐free erythroid differentiation of human pluripotent stem cells using small molecules. Stem Cells Transl Med. 2016;5(10):1394‐1405. 27400796 10.5966/sctm.2015-0371 PMC5031182

[55] Shah S , Huang X , Cheng L . Concise review: stem cell‐based approaches to red blood cell production for transfusion. Stem Cells Transl Med. 2014;3(3):346‐355. 24361925 10.5966/sctm.2013-0054 PMC3952923

[56] Ebrahimi M , Forouzesh M , Raoufi S , Ramazii M , Ghaedrahmati F , Farzaneh M . Differentiation of human induced pluripotent stem cells into erythroid cells. Stem Cell Res Ther. 2020;11(1):483. 33198819 10.1186/s13287-020-01998-9 PMC7667818

[57] Park YJ , Jeon S‐H , Kim H‐K , et al. Human induced pluripotent stem cell line banking for the production of rare blood type erythrocytes. J Transl Med. 2020;18(1):236. 32532292 10.1186/s12967-020-02403-y PMC7291485

[58] Posocco D , An H , Maguire JA , et al. Customized induced pluripotent stem cell‐derived red cell reagents. Blood. 2017;130:3.

[59] Jung HH , Danek A , Frey BM . McLeod syndrome: a neurohaematological disorder. Vox Sang. 2007;93(2):112‐121. 17683354 10.1111/j.1423-0410.2007.00949.x

[60] Hansen M , von Lindern M , van den Akker E , Varga E . Human‐induced pluripotent stem cell‐derived blood products: state of the art and future directions. FEBS Lett. 2019;593(23):3288‐3303. 31520530 10.1002/1873-3468.13599

[61] Chou ST . "Rare" reagent red cells: rare no longer? Transfusion. 2018;58(11):2469‐2471. 30284262 10.1111/trf.14958

[62] Shen J , Zhu Y , Lyu C , et al. Sequential cellular niches control the generation of enucleated erythrocytes from human pluripotent stem cells. Haematologica. 2020;105(2):e48‐e51. 31197070 10.3324/haematol.2018.211664 PMC7012464

[63] Sivalingam J , Lam A‐L , Chen HY , et al. Superior red blood cell generation from human pluripotent stem cells through a novel microcarrier‐based embryoid body platform. Tissue Eng Part C Methods. 2016;22(8):765‐780. 27392822 10.1089/ten.TEC.2015.0579

[64] Heshusius S , Heideveld E , Burger P , et al. Large‐scale in vitro production of red blood cells from human peripheral blood mononuclear cells. Blood Adv. 2019;3(21):3337‐3350. 31698463 10.1182/bloodadvances.2019000689 PMC6855111

[65] Trakarnsanga K , Griffiths RE , Wilson MC , et al. An immortalized adult human erythroid line facilitates sustainable and scalable generation of functional red cells. Nat Commun. 2017;8:14750. 28290447 10.1038/ncomms14750 PMC5355882

