# Supplementary material for: Generation of ‘designer erythroblasts’ lacking one or more blood group systems from CRISPR/Cas9 gene‐edited human‐induced pluripotent stem cells
Source: J Cell Mol Med. 2021 Sep 21;25(19):9340–9. doi: 10.1111/jcmm.16872 (PMC8500969; doi:10.1111/jcmm.16872)
Supplement: Supplementary file 1 — Fig S1 [file JCMM-25-9340-s002.pdf]

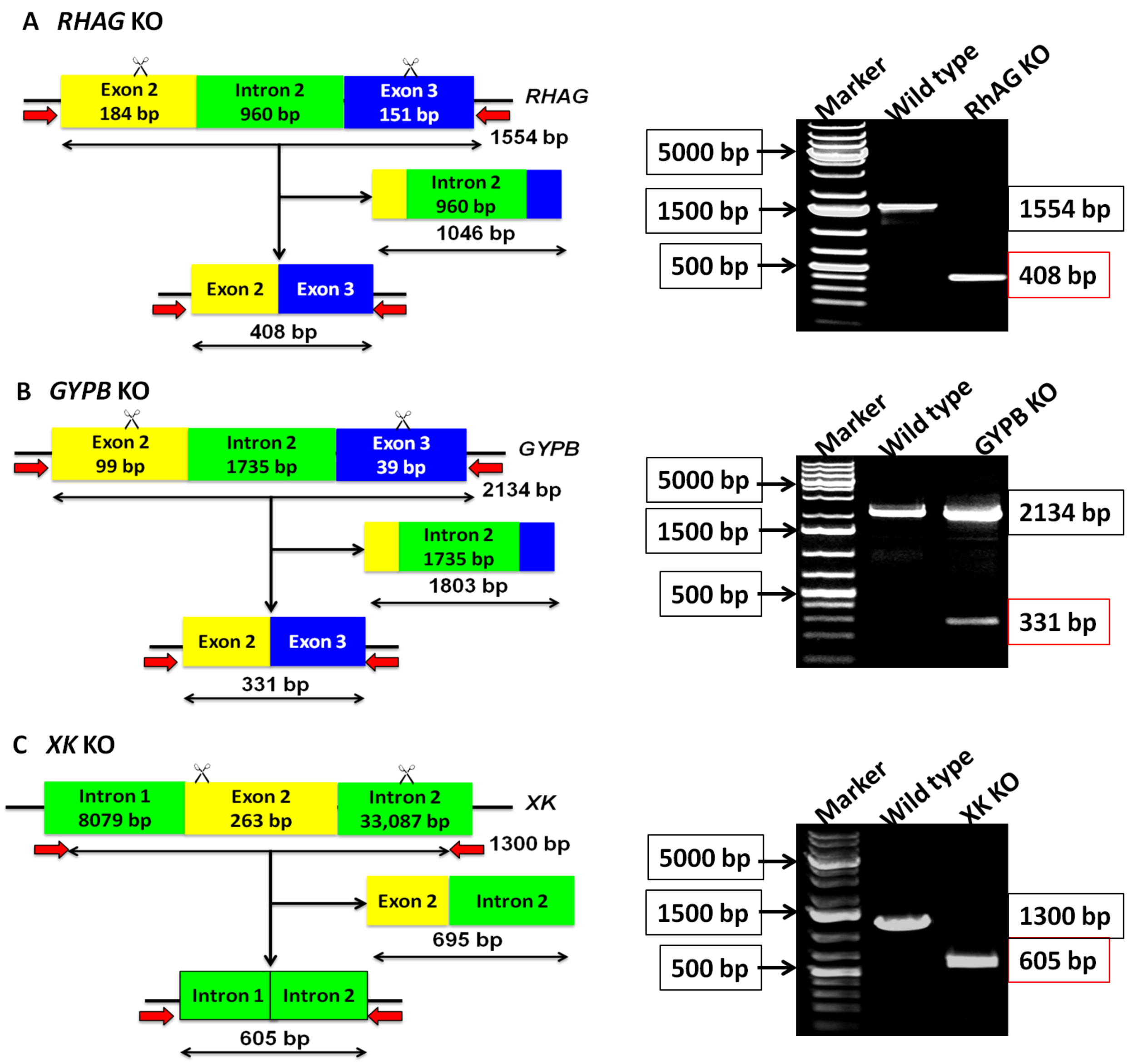

Supplementary Figure 1 Confirmation of CRISPR/Cas9 gene edited hiPSC knockout (KO) lines. Genotyping confirmed knockout of *RHAG* (A), *GYPB* (B), and *XK* (C) in the gene-edited KO cell lines. Right panel of the figure shows strategy adopted to delete the genes in question, where scissors represent the guide RNA positions and red arrows, genotyping primers. While complete knockout of *RHAG* and *XK* was observed, as demonstrated by the presence of a single 400 bp PCR band for the *RHAG* KO line and ~600 bp band for the *XK* KO line. Genotyping of *GYPB* KO demonstrated the presence of two bands: ~2100 bp (longer) and desired ~330 bp (shorter band). The presence of longer band in *GYPB* KO could be due to the working of only one guide RNA in some hiPSC creating an insertion/deletion (Indel), sufficient to generate a loss-of-function *GYPB* KO hiPSC line. The observation was confirmed by loss of GPB surface expression in *GYPB* KO derived erythroblasts. *RHAG*: Rh associated glycoprotein gene, *GYPB*: Glycophorin gene, *XK*: XK gene, GPB: Glycophorin B protein. Wild type: hiPSC parent line OT1-1
