# Supplementary material for: Generation of ‘designer erythroblasts’ lacking one or more blood group systems from CRISPR/Cas9 gene‐edited human‐induced pluripotent stem cells
Source: J Cell Mol Med. 2021 Sep 21;25(19):9340–9. doi: 10.1111/jcmm.16872 (PMC8500969; doi:10.1111/jcmm.16872)
Supplement: Supplementary file 2 — Table S1‐S2 [file JCMM-25-9340-s001.docx]

**Supplemental Table 1** Guide sequences finally used to knockout hiPSC

| **Gene** | **Genomic position** | **Guide Oligos** | **Sequence 5’ – 3’** | **PAM** |
| --- | --- | --- | --- | --- |
| *RhAG* | Exon 2 | Forward | CACCGCCTGTACAATAGTGCCCCAC | TGG |
|  |  | Reverse | AAACGTGGGGCACTATTGTACAGGC |  |
|  | Exon 3 | Forward | CACCGCACTAACCAGGTATTCATTG | TGG |
|  |  | Reverse | AAACCAATGAATACCTGGTTAGTGC |  |
| *XK* | Exon 2 | Forward | CACCGATGTCAGTATCACCAAGAAG | AGG |
|  |  | Reverse | AAACCTTCTTGGTGATACTGACATC |  |
|  | Intron 2 | Forward | CACCGTAAGTATTTCAACAATGAGA | AGG |
|  |  | Reverse | AAACTCTCATTGTTGAAATACTTAC |  |
| *GYPB* | Exon 2 | Forward | CACCGAGCATTAAGTACCACTGAGG | TGG |
|  |  | Reverse | AAACCCTCAGTGGTACTTAATGCTC |  |
|  | Exon 4 | Forward | CACCGGTCCATCGTTTCACTGTACC | AGG |
|  |  | Reverse | AAACGGTACAGTGAAACGATGGACC |  |
| **Annealing reaction set-up (10 µl)** | | | | |
| Guide Oligo Forward (100 µm) | | | 1 µl | |
| Guide Oligo Bottom (100 µm) | | | 1 µl | |
| T4 DNA ligation buffer (10X) | | | 1 µl | |
| Nuclease-free water | | | 7 µl | |
| **Annealing cycle** | | | | |
| **Temperature** | | **Time** | | |
| 37°C | | 10 minutes | | |
| 95°C | | 5 minutes | | |
| Ramp down to 25°C (0.1°C/second) | | | | |
| 10°C | | ∞ | | |

Post-annealing, gRNA were diluted with nuclease-free water in 1:100 ratio for subsequent use in gRNA-vector ligation reactions. Nucleotides marked in blue represents overhangs added to the guide sequences for cloning into BbsI digested Cas9 expression vector pX458/pX459V2. G-C nucleotides marked in red were added to the sequences to allow for transcription through U6 promoter. Oligos: oligonucleotide; PAM: protospacer adjacent motif

**Supplemental Table 2** Genotyping primer sequences and PCR cycle

| **Gene** | **Primer** | **Sequence 5’ – 3’** | | | **Fragment size** |  |
| --- | --- | --- | --- | --- | --- | --- |
| *RhAG* | Forward | GCTGGCATTGGAACAAGACTTT | | | 1554 bp |  |
|  | Reverse | TCTCACCAAGATTTGCTCCCA | | |  |  |
| *XK* | Forward | TTGCTTCAGTGGCCTTTGGT | | | 1300 bp |  |
|  | Reverse | AGACAGGAAAGTGGCCTTGTT | | |  |  |
| *GYPB* | Forward | TCTGCTCAGTCACCTCGTTC | | | 2132 bp |  |
|  | Reverse | CCTGGCCCCCAAAATGTTTTTA | | |  |  |
| **PCR reaction set-up (25 µl)** | | | | | | |
| DNA (50 ng/µl) | | | | 1 µl | | |
| Buffer (5X) | | | | 5 µl | | |
| Forward Primer (10 µM)  Reverse Primer (10 µM)  dNTP (25 mM)  DMSO  Herculase II Fusion DNA Polymerase | | | | 1 µl  1 µl  0.5 µl  0.5 µl  0.5 µl | | |
| Nuclease-free water | | | | 15.5 µl | | |
| **PCR cycle** | | | | | | |
| **Temperature** | | | **Time** | | | |
| 95°C | | | 2 minutes (initial denaturation) | | | |
| 95°C | | | 20 seconds | | | |
| Ta = 55°C (*RhAG* & *GYPA/B*), Ta = 64°C (*XK*) 30 cycles | | | | | | |
| 72°C  72°C  10°C | | | 1 minute  3 minutes (final amplification)  ∞ | | | |

Herculase II Fusion DNA Polymerase (Agilent Technologies, Santa Clara, CA) , dNTP: deoxynucleotide triphosphate
